# Immunological effects of nivolumab immunotherapy in patients with oral cavity squamous cell carcinoma

**DOI:** 10.1186/s12885-020-06726-3

**Published:** 2020-03-17

**Authors:** Ying Xiong, David M. Neskey, Joshua D. Horton, Chrystal M. Paulos, Hannah M. Knochelmann, Kent E. Armeson, M. Rita I. Young

**Affiliations:** 1 Department of Otolaryngology – Head and Neck Surgery, Medical University of South Carolina, Charleston, SC USA; 2 Hollings Cancer Center, Medical University of South Carolina, Charleston, SC USA; 3 Department of Cell and Molecular Pharmacology and Experimental Therapeutics, Medical University of South Carolina, Charleston, SC USA; 4 Department of Microbiology and Immunology, Medical University of South Carolina, Charleston, SC USA; 5 Department of Dermatology and Dermatologic Surgery, Medical University of South Carolina, Charleston, SC USA; 6 Research Service, Ralph H. Johnson Veterans Affairs Medical Center, Charleston, SC USA

**Keywords:** Anti-PD-1 antibody, CD4, CD8, Granzyme B, Immune, Interferon-γ, Nivolumab, T-cell

## Abstract

**Background:**

Although checkpoint blockades have become widely used, the immunological impact in cancer patients, especially those with oral cavity squamous cell carcinoma (OCSCC), has not been well studied.

**Methods:**

The present study assessed the immunological impact of anti-PD-1 (nivolumab) treatment in 10 patients with OCSCC. This involved phenotypic analyses of peripheral blood T-cell subpopulations and their expression of immune mediators prior to and following nivolumab treatment. The focus was on immunological effects of treatment without regard to possible clinical responses.

**Results:**

Nivolumab caused a decline in the frequency of blood CD4^+^ cells but did not affect their expression of IFN-γ. However, nivolumab increased the proportion of CD4^+^ cells expressing the Treg-supporting factor Foxp3. Nivolumab treatment caused an increase in the proportion of CD8^+^ cells. While their expression of granzyme B increased, it did not attain significance. Analyses of CD8^+^ cell subpopulations showed nivolumab caused an increase in levels of unconventional CD8^dim^CD3^+^ T-cells. It also caused an increase in expression of granzyme B by these unconventional T-cells as well as by the conventional CD8^hi^CD3^+^ cells. The CD8^hi^CD3^+^ subpopulation also had a near-significant increase in IFN-γ expression. Treatment with nivolumab had no effect on the levels of the NK containing CD8^dim^CD3^−^ subpopulation of cells or their expression of IFN-γ or granzyme B.

**Conclusions:**

These results show nivolumab causes opposing effects on CD4^+^ and CD8^+^ cell populations, with CD4^+^ cell levels declining but increasing the proportion of Treg cells, and unconventional CD8^+^ T-cell levels increasing with increased expression of immune mediators by CD8^+^ T-cell subpopulations.

## Background

Checkpoint inhibitors such as those targeting programmed cell death-1 (PD-1) are becoming widely used in cancer treatment regimens. The FDA has recently granted approval for use of anti-PD-1 antibodies for treatment of several cancer types including melanoma, non-small cell lung cancer (NSCLC), hepatocellular carcinoma, cervical cancer, Merkel cell carcinoma, and head and neck squamous cell carcinoma [1]. Regardless of whether antibodies against the PD-1/PD-L1 axis are used alone or in combination with other treatments, only a portion of patients respond to PD-1 inhibition [2–5]. Therefore, it is important to understand the immunological events that occur as a result of anti-PD-1 antibody treatment.

The rationale for treatment with antibodies to checkpoint inhibitors is the expectation that, by interrupting the immune inhibitory pathway, immune-mediated anti-cancer reactivity would be stimulated. However, relatively few studies have tested this premise in cancer patients. There is substantial indirect evidence of immune activation resulting from anti-PD-1 antibody treatment. Such evidence includes the immune-associated adverse events in patients receiving treatment. For example, anti-PD-1 antibody-treated patients with metastatic melanoma have shown immune-related diabetes conditions and increased levels of the inflammatory indicator C-reactive protein [6]. Metastatic melanoma patients have also been reported to experience thyroid (12% of subjects), rheumatological (22%) and dermatological (24% of subjects) dysfunctions [7]. Thyroid dysfunction associated with treatment of advanced melanoma patients with the anti-PD-1 antibody pembrolizumab was associated with increases in circulating CD56^+^CD16^+^ natural killer (NK) cells and increased HLA-DR expression on CD14^+^CD16^+^ monocytes compared to levels in healthy controls [8].

There have also been some direct analyses of the immunological effects of treatment with anti-PD-1 antibodies, although many of these are case reports on individual subjects. For example, a patient with metastatic NSCLC treated with pembrolizumab developed skin lesions containing infiltrating CD8^+^PD-1^+^ T-cells expressing the proliferation marker Ki-67 [9]. This was suggested to be indicative of reinvigorated T-cells within the anti-PD-1 antibody-induced skin rash. Similarly, an increase in Ki-67-expressing PD-1^+^CD8^+^ T-cells was seen after pembrolizumab treatment of melanoma patients, consistent with reinvigoration of exhausted T-cells [10]. A case report of a patient with head and neck squamous cell carcinoma described that treatment with the anti-PD-1 antibody nivolumab caused an increase in antibody production to the tumor antigen NY-ESO-1 and increased plasma cytokine and chemokine levels, although levels decreased with continued anti-PD-1 antibody treatment [11]. As a result of pembrolizumab treatment, a patient with metastatic melanoma brain lesions, was reported to have increased ratios of CD8:Treg cells and CD8:CD4 (non-Treg) cells in the blood [12]. Treatment of NSCLC patients with anti-PD-1 antibodies (pembrolizumab or nivolumab) expanded the levels of effector PD-1^+^ CD8 T cells expressing the costimulatory molecules CD28, CD27, and ICOS [13].

Few studies have assessed the immunological impact of anti-PD-1 (nivolumab) immunotherapy on peripheral blood T-cell subpopulations in patients with oral cavity squamous cell carcinoma (OCSCC). T-cell subpopulations include CD4^+^ T-helper or immune inhibitory T-regulatory (Treg) cells, as well as CD8^+^ cell subpopulations. Based on the level of expression of CD8 and/or other markers, several CD8^+^ subpopulations have been described. This includes conventional CD8^+^ cells, unconventional CD8^+^ cells associated with chronic infection and immune fatigue, and NK-containing populations of CD8^+^ cells [14–16]. In the present study, patients received nivolumab therapy prior to definitive surgical resection. The phenotypes of both CD4^+^ cells and CD8^+^ subpopulations, as well as their expression of immune mediators were assessed prior to and following anti-PD-1 antibody treatment.

## Methods

### Patient treatment

A single institution, single arm, Phase II clinical trial was conducted of neoadjuvant presurgical nivolumab (anti-PD-1 monoclonal antibody) for histologically proven, locoregionally advanced oral cavity squamous cell carcinoma (NCT03021993). This study was approved by the Institutional Review Board of the Medical University of South Carolina. Treatment with nivolumab was by intravenous injection of 3 mg/kg on day 1, 14 and 28. Based on preliminary clinical evaluation conducted between days 28 and 35, patients with progressive disease proceeded to definitive surgical resection. Patients with stabile disease or clinical response received a 4th nivolumab treatment on day 43 and then definitive surgical resection at approximately day 50–56. Blood was collected prior to initiating nivolumab treatment and at the time of surgery to determine how anti-PD-1 antibody treatment influences blood lymphoid cell subpopulations.

### Phenotypic analysis of T-cell subpopulations

Peripheral blood mononuclear cells (PBMC) from patients’ blood samples were isolated by Histopaque®-1077 density gradient centrifugation (Sigma, St Louis, MO). PBMCs were immediately cryopreserved so that pre- and post-treatment samples could be concurrently analyzed. Prior to antibody staining, cells were thawed and stimulated for 5 h at 37 °C with 50 ng/ml PMA, 1 μg/ml ionomycin, in the presence of 10.6 μM Brefeldin A and 2 μM Monensin (Invitrogen, Carlsbad, CA). Cell viability was based on forward and side scatter, and cells that appeared to be non-viable were excluded. A formal cell viability stain was not performed.

Fluorescence-conjugated mouse anti-human antibodies were all purchased from BD Biosciences (San Diego, CA). Cell surface expressions of CD3, CD4 and CD8 were determined using anti-CD3-APC-Cy7 (SK7), anti-CD4-PerCP-Cy5.5 (RPA-T4), and anti-CD8-PE (RPA-T8) antibodies. Cells were then fixed and permeabilized with Cytofix/Cytoperm kit (BD Biosciences). For determination of the intracellular levels of IFN-γ and granzyme B, anti-IFN-γ-FITC (B27) and anti-granzyme B-Alexa Fluor 647 (GB11) antibodies were used. For intracellular Foxp3 staining, the Human T17/Treg Phenotyping kit (BD Bioscience) was used according to the manufacturer’s instructions. Positively stained cells were quantitated using a FACSCanto flow cytometer (BD Biosciences).

### Statistical analyses

All patient samples were immunostained and flow cytometrically analyzed at least twice. The significance of differences between pre-treatment and post-treatment proportions of cell populations was analyzed by a paired Student’s t test as well as by a Wilcoxon signed-rank test, resulting in similar conclusions. Results of the paired Student’s t test are shown. Differences were considered to be statistically significant at a *p* value of < 0.05.

## Results

### Patient characteristics

From April 2017 to March 2019, 10 patients completed stage 1 of the nivolumab trial and were included in the current analysis. All included patients had squamous cell carcinoma of the oral cavity. Table 1 shows the characteristics of the OCSCC cancer patients that were enrolled in this study. All subjects received 3–4 treatments with anti-PD-1 antibody prior to definitive surgical treatment and there were no delays in definitive surgical treatment. Since the objective of this study was to assess the immunologic impact of anti-PD-1 therapy, the phenotypic analyses of peripheral blood leukocytes were analyzed independent of preliminary clinical evaluation.

**Table 1:** Enrollment patient characteristics

	All patients (*n* = 10)*
Age, years	
Mean ± SD	62.0 ± 7.3
Median (range)	60.5 (48–75)
Sex (%)	
Male	5 (50)
Female	5 (50)
Smoking status (%)	
Current	6 (60)
Former	2 (20)
Never	2 (20)
ECOG status (%)	
0	4 (40)
1	6 (60)
T stage (%)	
T2	3 (30)
T3	1 (10)
T4a	6 (60)
N stage (%)	
N0	4 (40)
N1	3 (30)
N2c	3 (30)
Clinical stage (%)	
II	3 (30)
IVA	7 (70)

* Of the 10 patients analyzed in this study 5 patients received 4 doses and 5 patients received 3 doses of nivolumab based on the trial design

### Effect of nivolumab treatment on CD4^+^ cells in the peripheral blood

**Fig. 1:**
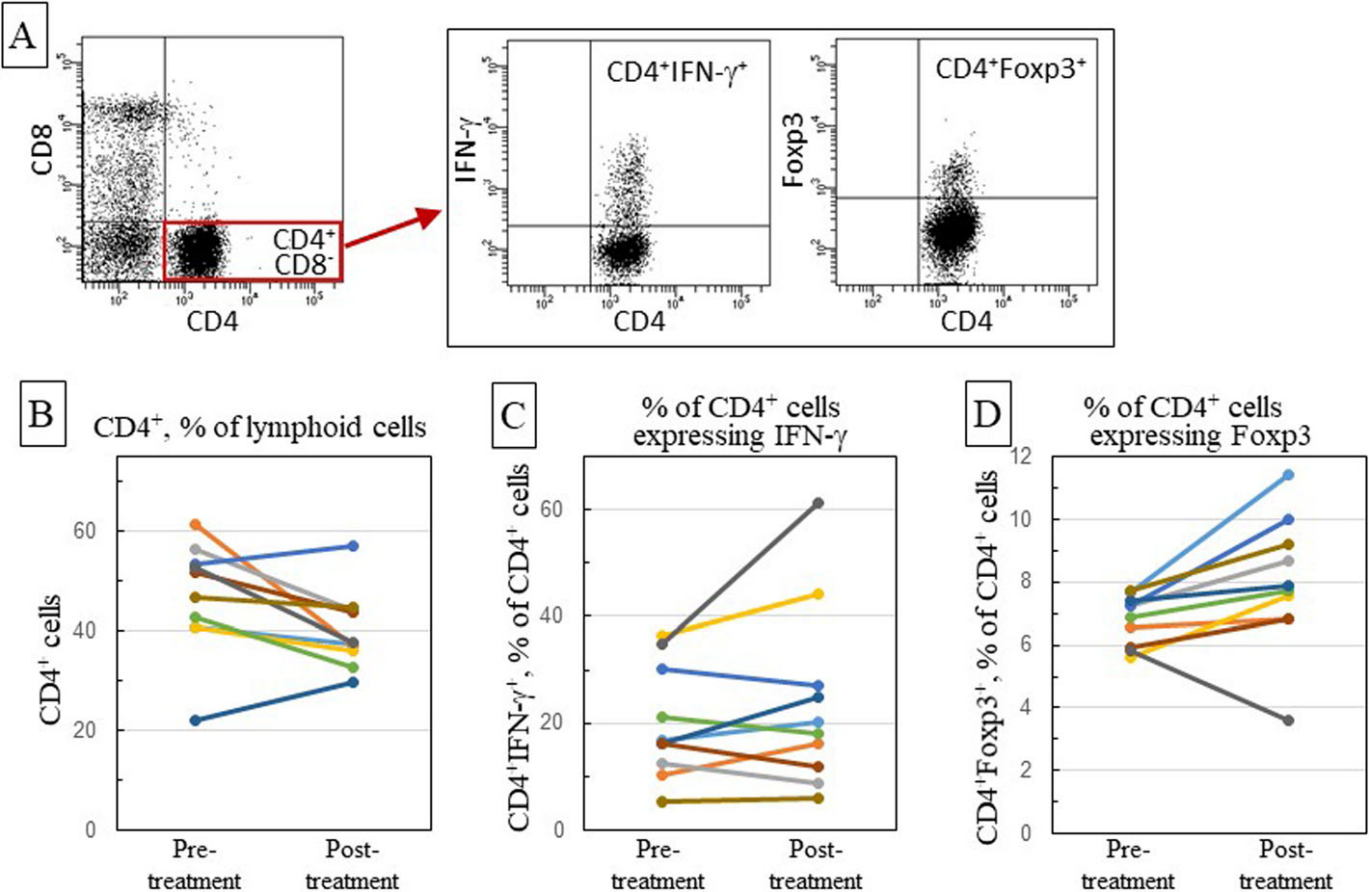
Effect of nivolumab treatment on levels of peripheral blood CD4^+^ cells, and their expression of immune mediators. Patient blood samples were collected prior to onset of nivolumab treatment and upon completion of treatment. Peripheral blood mononuclear cells (PBMC) were immunostained with antibodies to CD4, CD8, IFN-γ and Foxp3, and then analyzed by flow cytometry using the shown gating strategy (**a**). To quantitate the proportion of CD4^+^CD8^−^ cells (**a** and **b**), the analysis first gated on lymphoid cells. The CD4^+^CD8^−^ population was then gated on to determine the proportion of CD4^+^ cells that expressed IFN-γ (**a** and **c**) or Foxp3 (**a** and **d**). Each line color in panels **b**-**d** indicates the same patient across all panels

PBMC that were collected prior to and following treatment with anti-PD-1 antibody were first analyzed for levels of CD4^+^ T-cells and their expression of IFN-γ, IL-17 and Foxp3 using the gating strategy shown in Fig. 1a. Nivolumab caused a decline in blood levels of CD4^+^ T-cells (Fig. 1b, Table 2; *p* = 0.045). This decline was observed for 8 of the 10 patients that received nivolumab. There was not a statistically significant difference in the proportion of CD4^+^ T-cells that expressed IFN-γ between pre- and post-treatment samples (Fig. 1c, Table 2). Expression of IL-17 by CD4^+^ T-cells was low and did not change as a result of nivolumab treatment (not shown). Interestingly, nivolumab caused a significant increase in the levels of CD4^+^ T-cells expressing Foxp3 (*p* = 0.047), with 9 of 10 patients having an increased proportion of CD4^+^ cells expressing the Treg phenotype (Fig. 1d, Table 2).

**Table 2:** Summary of phenotypic analyses of patient leukocytes

	Pre-treatment	Post-treatment	*p*-value
CD4^+^ cells (%)	46.9	40.1	**0.045**
% of CD4^+^ cells			
IFN-γ ^+^	20.0	23.9	0.225
Foxp3^+^	7.7	9.2	**0.047**
CD8^+^ cells (%)	25.6	30.1	**0.043**
% of CD8^+^ cells			
IFN-γ ^+^	58.0	62.2	0.108
Granz B^+^	46.7	55.5	0.101
% of CD8^+^CD4^−^CD3^+^ cells			
CD8^dim^CD3^+^	10.4	12.2	**0.015**
CD8^dim^CD3^−^	27.4	28.7	0.709
CD8^hi^CD3^+^	61.6	59.1	0.454
% of CD8^dim^CD3^+^ cells			
IFN-γ^+^	60.7	62.9	0.404
Granzyme B^+^	34.7	42.6	**0.023**
% of CD8^dim^CD3^−^ cells			
IFN-γ^+^	70.5	68.9	0.572
Granzyme B^+^	91.2	91.5	0.853
% of CD8^hi^CD3^+^ cells			
IFN-γ^+^	51.1	57.3	0.054
Granzyme B^+^	26.4	36.7	**0.009**

Peripheral blood leukocytes were collected from OCSCC patients prior to and following treatment with nivolumab. Cells were immunostained flow cytometrically analyzed

### CD8^+^ cells and their effector cytokine profile prior to and following nivolumab

**Fig. 2:**
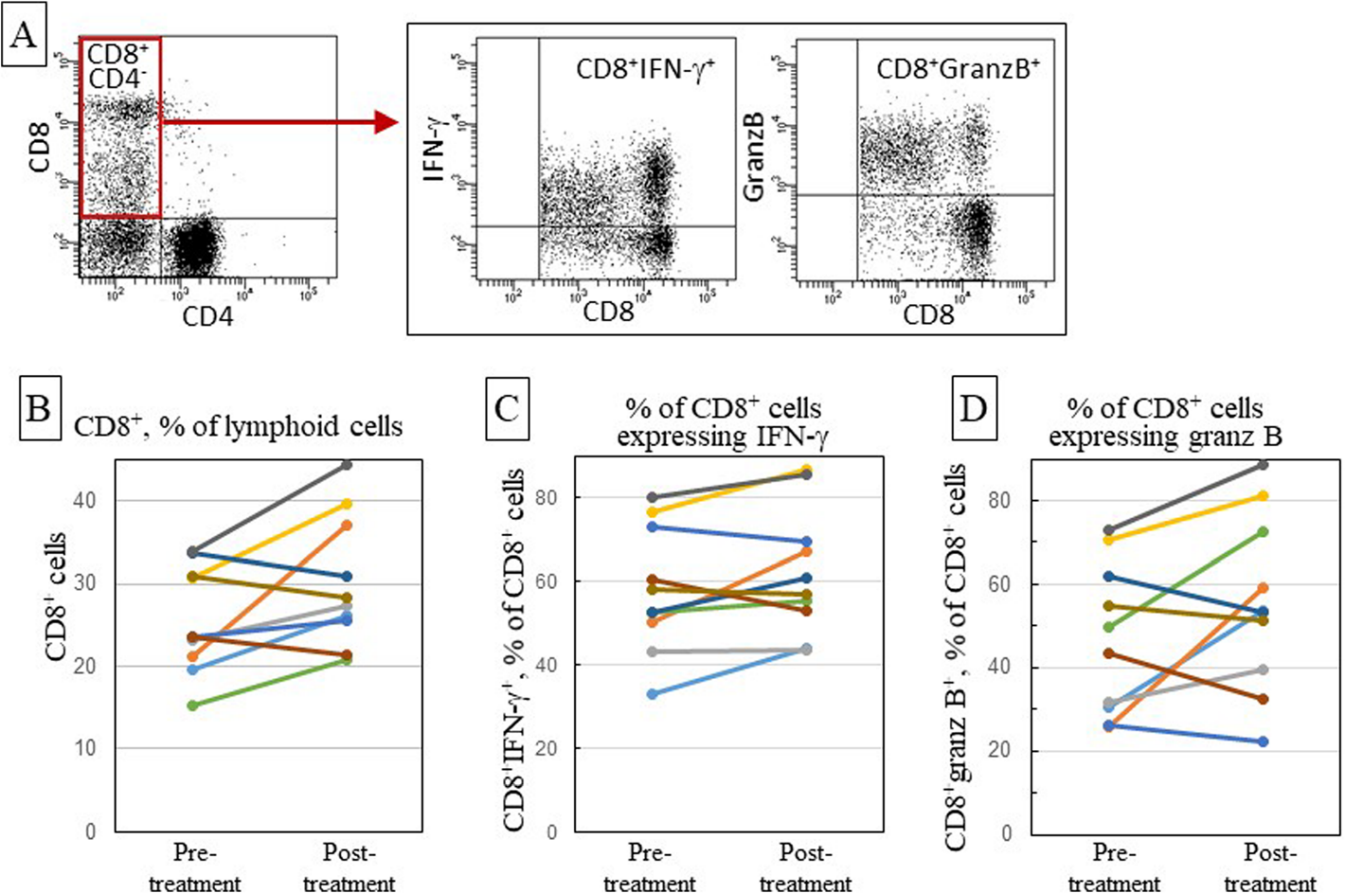
Effect of nivolumab treatment on levels of peripheral blood CD8^+^ cells, and their expression of immune mediators. Patient blood samples were collected prior to onset of nivolumab treatment and upon completion of treatment. PBMC were immunostained with antibodies to CD4, CD8, IFN-γ and granzyme B (GranzB), and then analyzed by flow cytometry using the shown gating strategy (**a**). To quantitate the proportion of CD8^+^CD4^−^ cells (**a** and **b**), the analysis first gated on lymphoid cells. The CD8^+^CD4^−^ population was gated on to determine the proportion of CD8^+^ cells that expressed IFN-γ (**a** and **c**) or granzyme B (**a** and **d**). Each line color in panels **b**-**d** indicates the same patient across all panels

The frequency of CD8^+^ cells and their expression of the cytokine IFN-γ and the cytotoxin granzyme B in PBMC of OCSCC patients was analyzed prior to and after nivolumab treatment. The gating strategy for analyzing CD8^+^ cell expression of IFN-γ and granzyme B is shown in Fig. 2a. Nivolumab treatment caused a significant increase in levels of CD8^+^ cells (*p* = 0.043; Table 2, Fig. 2b). The treatment caused this increase in blood levels of CD8^+^ cells in 7 of the 10 subjects. Levels of CD8^+^ cells expressing IFN-γ increased following treatment in 7 of the 10 patients, although this increase was not statistically significant (Table 2, Fig. 2c). In three of the subjects, levels of IFN-γ ^+^CD8^+^ cells declined. The proportion of CD8^+^ cells expressing granzyme B increased after anti-PD-1 treatment in 6 of the 10 patients, although the overall increase in CD8^+^ cells expressing granzyme B was not significant (Table 2, Fig. 2d).

### Analysis of CD8^+^ cell subpopulations

**Fig. 3:**
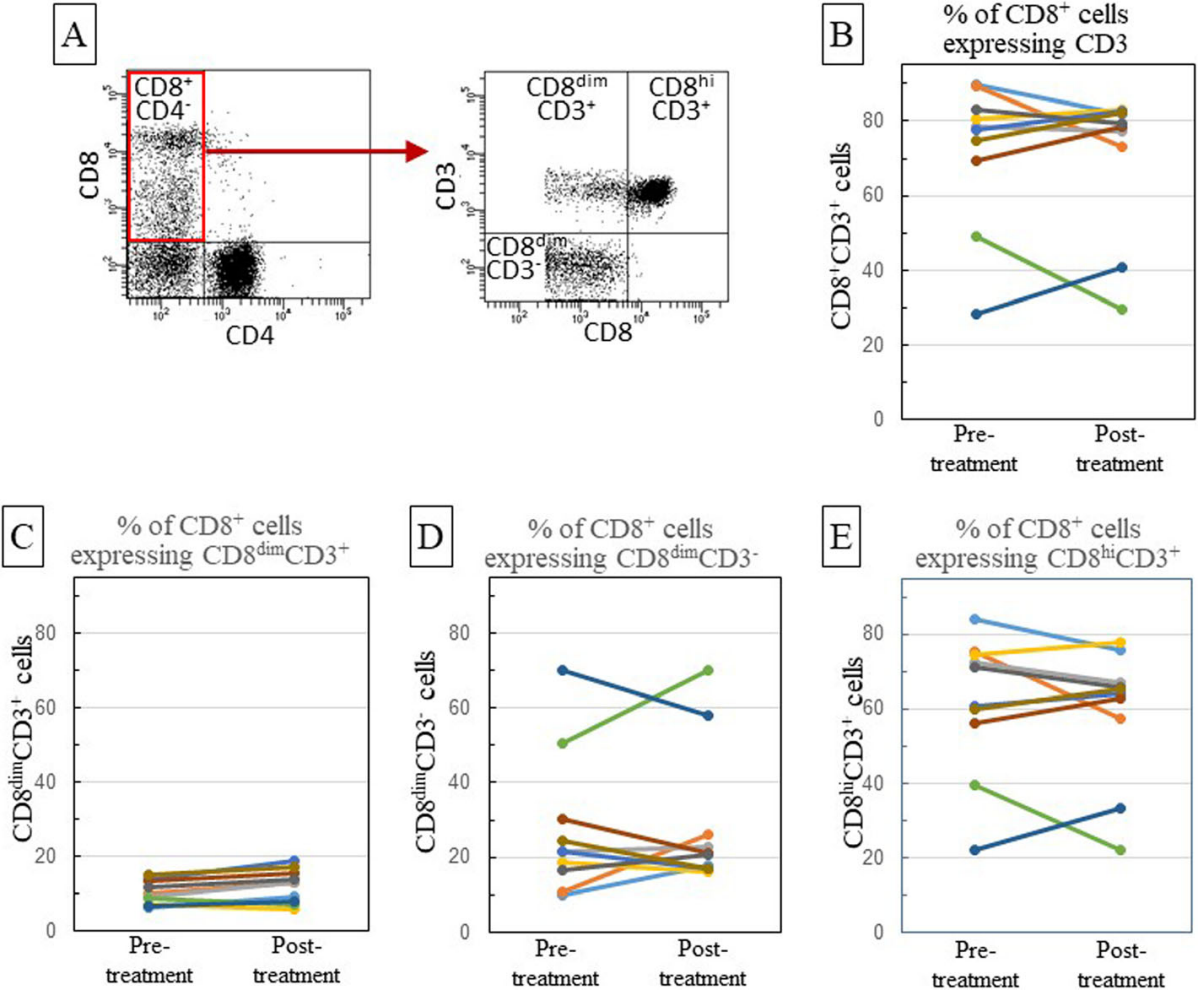
Analysis of CD8^+^ subpopulations. Blood leukocytes were immunostained for CD4, CD8 and CD3. CD8^+^CD4^−^ cells (**a**) were then identified as cells that expressed higher levels of CD8 plus CD3 (CD8^hi^CD3^+^), low levels of CD8 plus CD3 (CD8^dim^CD3^+^) or low levels of CD8 but not CD3 (CD8^dim^CD3^−^). The proportion of CD8^+^CD4^−^ cells that expressed CD3 pre and post nivolumab treatment for each patient is in panel **b**. In addition, CD8^+^ subpopulation analysis pre- and post-anti-PD-1 treatment was quantified for each OCSCC patient. Shown are the proportions of CD8^+^CD4^−^ cells that expressed CD3 and low levels of CD8 (**c**), low levels of CD8 but not CD3 (**d**) or expressed CD3 and high levels of CD8 (**e**). Each line color in panels **b-e** indicates the same patient across all panels

Since the aforementioned analyses showed an increase within the proportion of CD8^+^ cells in the peripheral blood following anti-PD-1 antibody treatment, a more elaborated analysis of CD8^+^ cell subpopulations was conducted. Fig. 3a is a typical scatter plot showing two distinct subpopulations of cells staining negative for CD4 however staining positive for CD8, with one subpopulation stained bright for CD8 and the other more dimly. To further characterize these cells, they were co-stained with CD3 antibody (Figs. 3 a and b, Table 2), which disclosed three CD8^+^ cell subpopulations: cells staining bright for CD8 and staining for CD3 (CD8^hi^CD3^+^), cells staining more dimly for CD8 and staining for CD3 (CD8^dim^CD3^+^) and cells staining dimly for CD8 but not staining for CD3 (CD8^dim^CD3^−^).

The proportions of each of these three CD8^+^ subpopulations varied among patients, with CD8^hi^CD3^+^ being the majority of CD8^+^ T-cells in 8 of the 10 patients (Fig. 3e). In 2 subjects, the majority of the CD8^+^ cells were CD8^dim^CD3^−^, a population that would likely include NK cells (Fig. 3d). Although the total frequency of CD8^+^ cells in the peripheral blood increased following nivolumab treatment, in most cases the relative proportions of these three CD8^+^ subpopulations following treatment was not statistically different from the pre-treatment levels. The exception was CD8^dim^CD3^+^ T-cells. The proportion of CD8^+^ T-cells that were CD8^dim^CD3^+^ cells was low, but nivolumab treatment caused an increase in the proportion of this subpopulation (*p* < 0.015), with 8 of the 10 patients showing increased levels (Table 2, Fig. 3c). The proportion of CD8^+^ T-cells that were CD8^hi^CD3^+^ declined in 5 of the 10 subjects but increased in the other 5 subjects (Fig. 3e). The effect of anti-PD-1 antibody treatment on the proportion of CD8^+^ cells that were CD8^dim^CD3^−^ varied similarly among subjects, with 5 of the 10 subjects having an increased proportion of these cells and 5 of the subjects having a reduced proportion following treatment (Table 2, Fig. 3d). The patients whose proportion of CD8^hi^CD3^+^ cells was reduced by nivolumab treatment were the same as the patients whose proportion of CD8^dim^CD3^−^ cells increased. Overall, these results show broad variation in the effect of nivolumab treatment on CD8^+^ cell subpopulations with the most consistent being an increase in the proportion of CD8^dim^CD3^+^ cells.

### IFN-γ and granzyme B expression by peripheral blood CD8^+^ cell subpopulations

**Fig. 4:**
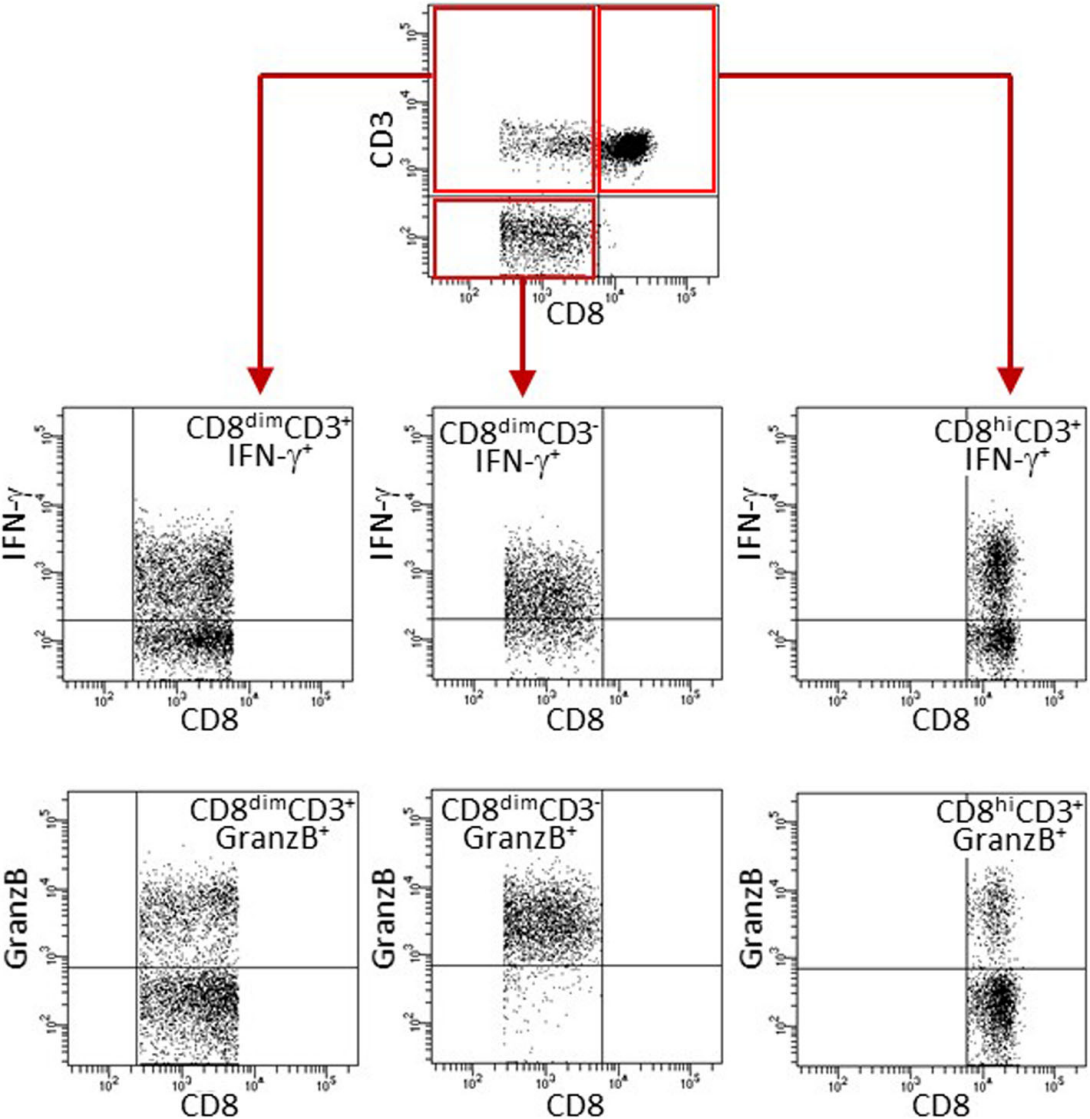
Gating strategy for analysis of expression of IFN-γ or granzyme B by CD8^+^ subpopulations. Blood leukocytes were immunostained for CD4, CD8 CD3, IFN-γ or granzyme B. CD8^+^CD4^−^ cells were then identified as cells that expressed higher levels of CD8 plus CD3 (CD8^hi^CD3^+^), low levels of CD8 plus CD3 (CD8^dim^CD3^+^) or low levels of CD8 but not CD3 (CD8^dim^CD3^−^). Each of these subpopulation was then gated on and analyzed for their expression of IFN-g or granzyme B

**Fig. 5:**
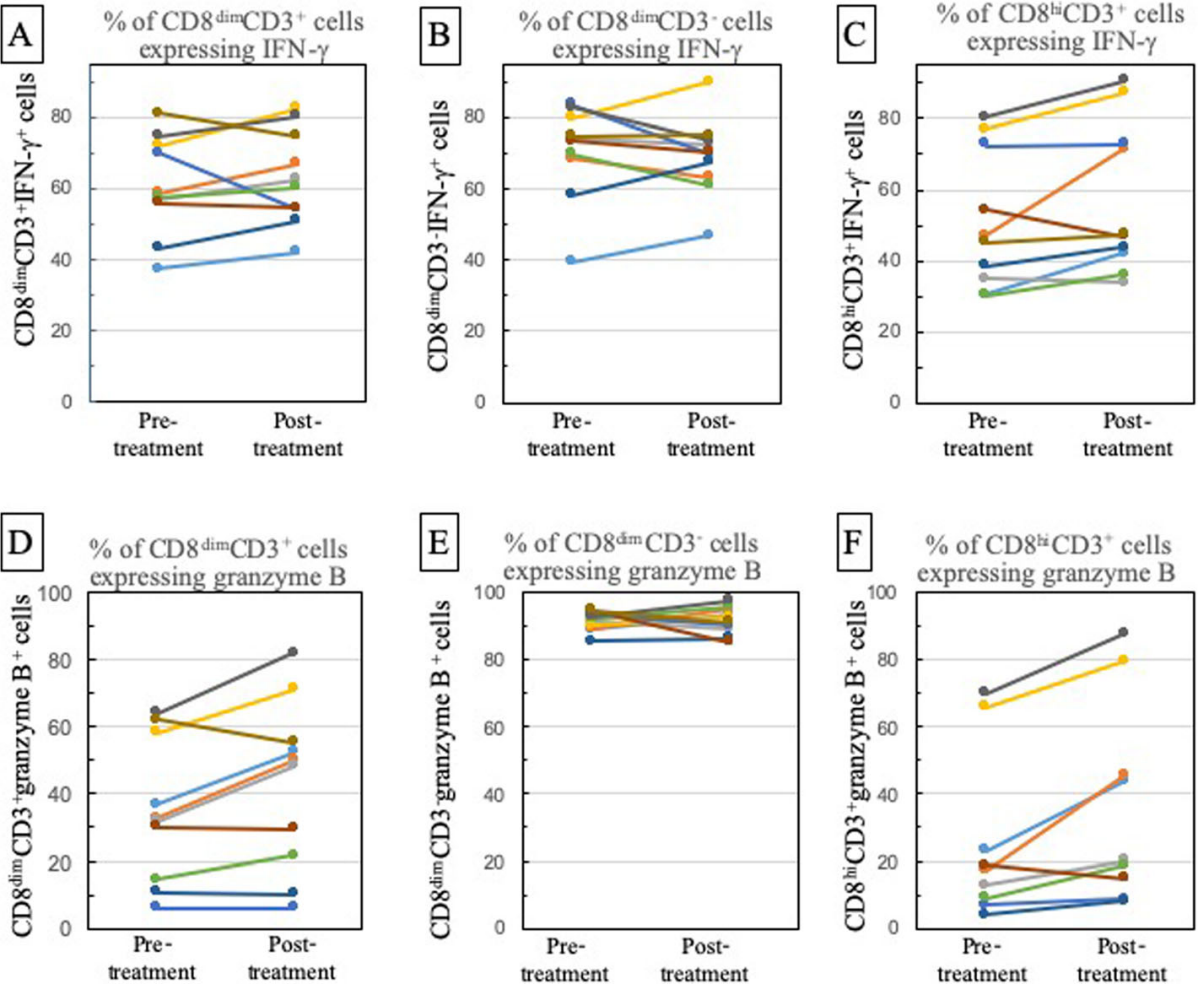
Effect of nivolumab on expression of IFN-γ and granzyme B by peripheral blood CD8^+^ cell subpopulations. Patient blood samples that were collected prior to and after completion of nivolumab treatment were immunostained with antibodies to CD8, CD4, CD3, IFN-γ, and granzyme B. The proportion of CD8^dim^CD3^+^ cells (**a**), CD8^dim^CD3^−^ (**b**) or CD8^hi^CD3^+^ (**c**) that expressed IFN-γ prior to and following treatment is shown for each OCSCC patient. The proportion of CD8^dim^CD3^+^ cells (**d**), CD8^dim^CD3^−^ (**e**) or CD8^hi^CD3^+^ (**f**) that expressed granzyme B prior to and following treatment is shown for each OCSCC patient Each line color indicates the same patient across all panels

The proportion of CD8^+^ cell subpopulations that expressed IFN-γ or granzyme B was assessed prior to and following treatment of OCSCC patients with nivolumab. The gating strategy for these flow cytometric analyses is shown in Fig. 4. Nivolumab treatment did not cause a change in the proportion of CD8^dim^CD3^+^ or CD8^dim^CD3^−^ subpopulations that expressed IFN-γ (Table 2, Figs. 5a and 3b). However, nivolumab increased the proportion of CD8^dim^CD3^+^ T-cells that expressed IFN-γ in 7 of the 10 subjects (Fig. 5a). In contrast, the proportion of CD8^dim^CD3^−^ cells (NK-containing population) that expressed IFN-γ only increased following treatment in 4 of the 10 subjects (Fig. 5b). Although not quite reaching statistical significance (*p* = 0.054), there was a strong trend toward an increase in the proportion of CD8^hi^CD3^+^ T-cells that expressed IFN-γ following nivolumab treatment (Table 2, Fig. 5c). Nivolumab increased the proportion of CD8^hi^CD3^+^ T-cells that expressed IFN-γ in 7 of the 10 patients. The mean fluorescence intensity of IFN-γ expression by the CD8^+^ subpopulations was also analyzed, but there were no differences between pre- and post-treatment in the intensity IFN-γ expression by any of these three CD8^+^ subpopulations (data not shown).

Expression of granzyme B by the three CD8^+^ cell subpopulations was also examined. Nivolumab treatment significantly increased the proportion of CD8^hi^CD3^+^ cells that expressed granzyme B (Table 2, Fig. 5f; *p* = 0.009). In 9 of the 10 subjects, the proportion of CD8^hi^CD3^+^ cells that expressed granzyme B increased. In addition, nivolumab treatment significantly increased the proportion of CD8^dim^CD3^+^ cells that expressed granzyme B (Table 2, Fig. 5d; *p* = 0.023). This increase was seen for 6 of the 10 patients, with no change in the proportion of CD8^dim^CD3^+^ cells expressing granzyme B in 3 of the subjects and a decline in levels of this subpopulation in 1 patient. Most of the patients that had an increase in the proportion of CD8^dim^CD3^+^ cells expressing granzyme B also had an increase in CD8^hi^CD3^+^ cells expressing granzyme B. Almost all of the CD8^dim^CD3^−^ subpopulation expressed granzyme B prior to onset of anti-PD-1 antibody treatment and this remained unchanged following treatment (Table 2, Fig. 5e). These studies indicate that nivolumab treatment of OCSCC patients results in a tendency toward an increase in IFN-γ expression, particularly by the most predominant CD8^hi^CD3^+^ cell population, and causes a clear increase in granzyme B expression by the two CD8^+^CD3^+^ T-cell subpopulations (CD8^dim^CD3^+^ and CD8^hi^CD3^+^ cells).

## Discussion

The use of checkpoint blockades either alone, in combination, or with other forms of treatment for cancer patients has become more widely used [2–5, 7, 8]. However, the immunological effect of checkpoint blockades has not been extensively studied in cancer patients and, in particular, OCSCC patients. The objective of this study was to assess the immunological effect in the peripheral blood of blocking the PD-1/PD-L1 axis by treating patients with surgically resectable OCSCC with antibody to PD-1.

The results of this study showed that nivolumab caused a reduction in blood levels of CD4^+^ T-cells in OCSCC patients but did not affect the proportion of CD4^+^ T-cells that expressed IFN-γ. In contrast to the lack of nivolumab treatment effect on CD4^+^ cell expression of IFN-γ, nivolumab caused an increase in the proportion of Foxp3^+^CD4^+^ T-cells. Expression of Foxp3 characterizes regulatory T-cells and would not have been expected to increase as a result of nivolumab treatment. The significance of the demonstration that nivolumab causes an increase in the proportion of CD4^+^ cells that expressed Foxp3 within the peripheral blood compartment remains unclear since most of the existing literature has focused on CD4^+^FoxP3^+^ population within the tumor infiltrating lymphocytes. In this setting increased CD4^+^FoxP3^+^ expressing T cells following PD-1 inhibition are associated with hyperprogression in patients with gastric cancer [17]. However, it is consistent with a study in which patients with NSCLC showed increased expression of a different checkpoint, killer cell Ig-like receptor, following nivolumab treatment, suggesting compensatory expression of immune checkpoints following PD-1 blockade [18].

Assessment of the effect of nivolumab therapy on CD8^+^ cells in the peripheral blood showed that nivolumab causes an increase in the levels of CD8^+^ cells and resulted in a near significant increase in their expression of IFN-γ and granzyme B. Moreover, 3 subpopulations of CD8^+^ cells were identified based on the intensity of CD8 expression and whether or not the cells co-expressed CD3. Although increases in IFN-γ expression were not statistically significant, nivolumab caused an increase in the proportions of the T-cell subpopulations expressing IFN-γ in most of the OCSCC patients suggesting an increase in the cytotoxic potential of CD8^+^ cells. Additionally CD8^+^ cells expressing IFN-γ may facilitate the migration of cytotoxic CD8 T lymphocytes (CTL) into the primary tissue as suggested by previous work in keratinocyte models that demonstrated IFN-γ derived from CTL directly enhances their cytotoxicity and motility to the target tissue [19]. The CD8^hi^CD3^+^ T-cell subpopulation was the most prominent CD8^+^ cell subpopulation in most patients. The proportion of these conventional T-cells that had an increase in IFN-γ expression following nivolumab treatment approached significance. Nivolumab treatment caused a significant increase in the proportion of CD8^hi^CD3^+^ T-cells expressing granzyme B, possibly indicating increased cytolytic activity. The levels of the unconventional CD8^dim^CD3^+^ T-cells expressing IFN-γ increased in most patients following anti-PD-1 therapy but did not reach significance. However, nivolumab treatment resulted in a significant increase in the proportion of these CD8^dim^CD3^+^ T-cells expressing granzyme B. The proportion of CD8^dim^CD3^−^ cells, which likely includes NK cells, and their expression of IFN-γ or granzyme B did not change between pre- and post-treatment levels.

Of interest was the increase in CD8^dim^CD3^+^ T-cells and their expression of granzyme B that was caused by nivolumab treatment. It was beyond the scope of the present study to conduct detailed phenotyping to assess the identity of these putative unconventional T-cells, but levels of CD8^dim^CD3^+^ T-cells have been shown to be increased due to high pathogen burdens or latent virus infections, which has been suggested to be indicative of immune exhaustion since greater than 80% of the CD^dim^ cells had an effector phenotype. Although these cells may be more differentiated the increased granzyme B expression clearly suggestion enhanced cytolytic activity [14, 15].

Only a few studies have conducted immunological analyses of cancer patients receiving anti-PD-1 antibody treatment. Although not significant, one study suggested that there was a tendency toward decreased levels of CD3^+^CD4^+^ T helper cell and increased levels of CD3^+^CD8^+^ T-cells in the peripheral blood of patients with advanced metastatic tumors responding to anti-PD-1 antibody [20]. Our study similarly showed that nivolumab caused a decrease in CD4^+^ T-cells in 7 of the 9 patients. Additionally, the present study showed that nivolumab similarly causes a significant increase in blood levels of CD8^+^ cells. Although the overall levels of CD8^+^ cells increased following treatment, nivolumab treatment only caused an increase in the relative proportion in the unconventional CD8^dim^CD3^+^ subpopulation, with the relative proportion of the conventional (CD8^hi^CD3^+^) and NK-containing (CD8^dim^CD3^−^) CD8^+^ cell subpopulations remaining constant.

Overall, the results of this immunological study showed anti-PD-1 antibody treatment of OCSCC patients does not cause an overall immune enhancing effect but, instead, increases levels of CD4^+^ Treg while stimulating CD8^+^ T-cell responses. These stimulatory responses prominently included nivolumab increasing blood levels of CD8^+^ T-cells, and increasing levels of T-cell subpopulations expressing IFN-γ and granzyme B in most of the OCSCC patients. This finding was particularly evident in the CD8^+^ subpopulations that contained T-cells (CD8^hi^CD3^+^ and CD8^dim^CD3^+^) as opposed to the subpopulation that likely included NK cells (CD8^dim^CD3^−^). These analyses should be continued with a larger number of patients together with definitive clinical evaluation of treatment effectiveness so as to enable stratification by subjects by those that showed clinical responses to nivolumab treatment versus those whose OCSCC progressed.

## Conclusion

Although checkpoint blockades have become widely used, their immunological impact in patients with oral cancer has not been well studied. The results of the present study showed that nivolumab treatment of patients having OCSCC causes opposing effects on CD4^+^ and CD8^+^ cell populations. Nivolumab stimulated CD8^+^ T-cell subpopulations in the peripheral blood. There was a tendency toward an increase in IFN-γ expression by CD8^+^ T-cell subpopulations, but nivolumab caused a prominent increase in their granzyme B expression. However, nivolumab treatment did not result in overall immune stimulation as it decreased the proportion of CD4^+^ cells, had no effect on their expression of IFN-γ, but increased their expression of the Treg marker Foxp3. The impact of this study is the demonstration that treatment of an understudied oral cancer population with nivolumab causes a stimulatory effect mainly on CD8^+^ cell subpopulations, rather than an expected overall immune stimulatory effect.

## Data Availability

The datasets used and/or analyzed during the current study are available from the corresponding author on reasonable request.

## Abbreviations

CTL: Cytotoxic CD8 T lymphocytes
NK: Natural killer
NSCLC: Non-small cell lung cancer
OCSCC: Oral cavity squamous cell carcinoma
PBMC: Peripheral blood mononuclear cells
PD-1: Programmed cell death-1
Treg: T-regulatory
